# Effect of Fibre Orientation and Hostile Solutions on Stress Relaxation of Glass/Polyamide Composites

**DOI:** 10.3390/polym12010020

**Published:** 2019-12-20

**Authors:** Paulo Nobre Balbis dos Reis, Ana Martins Amaro, Maria Augusta Neto

**Affiliations:** 1 C-MAST, Department of Electromechanical Engineering, University of Beira Interior, Calçada Fonte do Lameiro, 6201-100 Covilhã, Portugal; 2 CEMMPRE, Department of Mechanical Engineering, University of Coimbra, 3030-788 Coimbra, Portugal; ana.amaro@dem.uc.pt (A.M.A.); augusta.neto@dem.uc.pt (M.A.N.)

**Keywords:** thermoplastic matrix, hostile environments, viscoelastic behaviour, stress relaxation

## Abstract

Polyamide creates high-performance composite materials, which are replacing the traditional epoxy composites in several applications. In this context, exposure to hostile environments is expected. On the other hand, due to the viscoelastic nature of the matrix, these composite materials are prone to stress relaxation. Therefore, the stress relaxation behaviour of glass/polyamide 6 composites was studied considering different fibre directions, as well as exposure to NaOH and HCl solutions. Stress relaxation tests on the bending mode were carried out, and the stress recorded during the loading time (7200 s). All tests were characterized by a stress decrease over time, but laminates with higher fibre angles were more prone to stress relaxation. However, exposure to hostile solutions promoted more significant decreases, where the highest stress relaxation was achieved for alkaline environments with values that were three times higher for laminates with fibres at 0° and around one and half times higher for 45° fibre alignments when compared with the control samples. Finally, the Kohlrausch–Williams–Watts (KWW) model showed that it can be used to predict stress relaxation time, due to the accuracy that was obtained between the experimental and theoretical results.

## 1. Introduction

Composite materials with a polymeric matrix are becoming very common due to their excellent mechanical properties. Among the two types of polymeric matrices that can be identified, thermosets and thermoplastics, the latter one allows for faster processing techniques and much shorter cycle times [1].

Polyamide, for example, is an important engineering plastic, with excellent physical and mechanical properties, that creates high-performance composites [2]. Arhant et al. [3] studied the replacement of carbon/epoxy composites with carbon/polyamide composites for underwater applications, because thermoset-based composites (polyester/epoxy) require high thicknesses (more than 10 mm) and reaching such thicknesses without defects is a challenge. On the other hand, residual stresses and delaminations are common, and they could lead to premature failures. Despite the benefits obtained with polyimide laminates, they can microcrack spontaneously when exposed to hostile environments [4]. Han and Nairn [4], for example, observed that polyimide matrix composites present degradation in toughness when exposed to water and high temperature, with consequent microcracking. In fact, several studies can be found in the literature involving such composites, but many of them analysed their mechanical performance only when exposed to wet environments, seawater and/or high temperatures [4,5,6,7,8,9,10].

On the other hand, in terms of corrosive environments (alkaline and acidic solutions), the literature is much more abundant with respect to thermoset composites than thermoplastics. For instance, studies developed by Stamenovic et al. [11] in glass–polyester composites demonstrated that alkaline solutions are responsible for the decrease of tensile strength and elastic modulus, because they are highly corrosive, whereas the opposite tendency occurs when these composites are exposed to acid solutions. However, for both solutions, the changes observed are very dependent on the pH value and exposure time. Feng et al. [12] studied the effect of different solutions (H_2_SO_4_, NaCl and NaOH) on glass fibre-reinforced polymer (GFRP)/epoxy composites and observed a decrease in hardness, flexural strength and elastic modulus, and this trend is even higher for higher concentrations. Moreover, Amaro et al. [13] analysed the effect of alkaline (NaOH) and acid (HCl) solutions on the flexural properties of these composites, and the worst bending properties were obtained when the glass/epoxy composites were exposed to NaOH solutions. The effect of hydrochloric acid (HCl) and sulphuric acid (H_2_SO_4_) was also compared by these authors, and the HCl solution was responsible for the poorest results [14]. Similar conclusions were also obtained by Kamal and Kadhim [15] on nano-silica-reinforced (glass/Kevlar) fabric polyester hybrid composites, due to the higher corrosive effect of alkaline solutions. Finally, the effects of strong acids (HCl, H_2_SO_4_, HNO_3_ and H_3_PO_4_) on mechanical properties of glass/polyester glass-reinforced pipes (GRP) at normal and high temperatures were evaluated by Mahmoud and Tantawi [16]. They observed significant changes in terms of flexural strength, hardness and Charpy impact strength, depending on the period of immersion and the type of acid, as well as the immersion temperature.

Literature reports that polymer ageing can be classified as physical and chemical. Physical ageing can change the molecular conformation and cause embrittlement, whereas chemical ageing produces irreversible degradation of the molecular structure [17]. In this context, Benmokrane et al. [18] observed that both H_2_O and H^+^ are soaked through microcracks and voids, causing greater defects, and in particular, H^+^ reacted with the resin/fibre interfaces, promoting its debonding. These results seem to indicate that the damage mechanisms that are initiated by physical and/or chemical reactions between fibres and matrix are indeed responsible for the lower mechanical properties observed. In fact, Bazli et al. [19] reported that when the solutions penetrate through voids, cracks or interface fibre/matrix, an ion exchange is observed that, simultaneously, affects the matrix (chains scission) and interfacial fibre/matrix bond strength and promotes a decrease of the mechanical properties. Plain-weave laminates of C-glass and amine-cured epoxy exposed to water and sulfuric acid solutions were studied by Tanks et al. [20], and they also concluded that both solutions attack the fibre surface, but the sulfuric acid increases the saturation uptake of water in the material, causing high swelling stresses that significantly reduce mechanical properties. The resin/fibre interface degradation, due to the physical and chemical interactions, was also the main damage mechanism that explained the lower mechanical properties obtained by Wang et al. [21] on basalt composites exposed to acidic environments and also those obtained by Kusano et al. [22] on fibre-reinforced plastic tanks immersed in HCl solutions.

This literature review clearly highlights the importance of studying the effects of hostile environments on the mechanical performance over a composite materials lifetime [23]. However, due to the viscoelastic behaviour of polymers, polymeric composites are prone to creep and stress relaxation, which is a great challenge when they are used in long-term applications [24,25,26]. There are several published studies related to the creep effects on composites [27,28,29,30,31], inclusively related to harsh environments [27,32,33,34,35,36], but they are essentially focused on thermoset composites. On the other hand, literature is not as rich with respect to studies of stress relaxation, but, regardless of the matrix, some studies reported a significant decrease in stress over time [37,38,39,40]. Moreover, they also reported that this behaviour is strongly affected by the fibre orientation [37,41,42,43,44], temperature [37,41,42,44] and environment [45]. In fact, Amaro et al. [13,14] and Reis et al. [45] observed a significant decrease on the flexural strength and higher stress relaxation due to fibre/matrix interface degradation that was promoted by the immersion of the composite into acid and alkaline solutions. Hence, it is clear that the interface properties play a relevant role on stresses, because relaxation occurs due to the breaking of bonds and their propagation [46].

However, to the best of our knowledge, in the literature, there is a lack of studies about the effect of hostile solutions combined with different fibre directions on viscoelastic behaviour. Therefore, the main goal of the present study is to investigate the stress relaxation behaviour of glass/polyamide 6 composites, and for this purpose, alkaline (NaOH) and acid (HCl) solutions were selected. Stress relaxation tests were carried out on the bending mode, and the stress was recorded during the loading time. The bending mode was selected because, according to Banna et al. [47], it is the most sensitive for this type of analysis and one of the most popular with structural loading. Finally, as reported by Arhant et al. [3], the relevance of the present study is based on the replacement of composites of thermoset resins by thermoplastic ones, especially, for harsh environmental applications.

## 2. Materials and Methods

All samples were obtained from thin plates of continuous glass fibre-reinforced polyamide 6, supplied by Bond-Laminates GmbH (Brilon, Germany) under the trade name of Tepex^®^ dynalite 102-RG600(x)/47%. These plates had a 3 mm thickness and were cut using a diamond saw, and a moving speed chosen to reduce the heat on specimens, with dimensions of 60 mm × 10 mm (mm^2^). Finally, they were split into control and study groups, according to Table 1.

Three-point bending (3PB) static tests were performed with a spam of 48 mm, according to American Society for Testing and Materials (ASTM) Standard D 7264/D 7264M-07 [48], and a Shimadzu AG-10 (Riverwood Drive Columbia, USA) universal testing machine equipped with a 5 kN load cell was used. For each condition, at least five specimens were tested at room temperature and at a rate of 3 mm/min.

The flexural strength was calculated as the nominal stress at the middle span section and evaluated using the maximum value of the load (Equation (1)), while the bending stiffness modulus was obtained by linear regression of the load–displacement curves considering the interval in the linear segment with a correlation factor greater than 95% according to Equation (2) [49]:

(1) $$\sigma =\frac{3\;P\;L}{2\;b\;h^{2}}$$

(2) $$E=\frac{\Delta P\cdot L^{3}}{48\Delta u\cdot I}$$

where *P* is the load, *L* the span length, *b* the width, *h* the thickness of the specimen, *I* the moment of inertia of the cross section and Δ*P* and Δ*u*, respectively, the load range and flexural displacement range in the middle span for an interval in the linear region of the load versus displacement plot.

Stress relaxation (SR) tests were also performed with the same equipment (Shimadzu AG-10, Riverwood Drive Columbia, USA), at room temperature and with geometry similar to those used on the bending tests. All experimental procedures were supported by ASTM E328-02 [50], where a fixed strain was applied (corresponding to around 30 MPa for all configurations) and the stress recorded during the loading time of 7200 s. This bending stress value was selected to guarantee that all SR tests were carried out in the elastic regime of all conditions studied.

## 3. Results and Discussion

**Figure 1:**
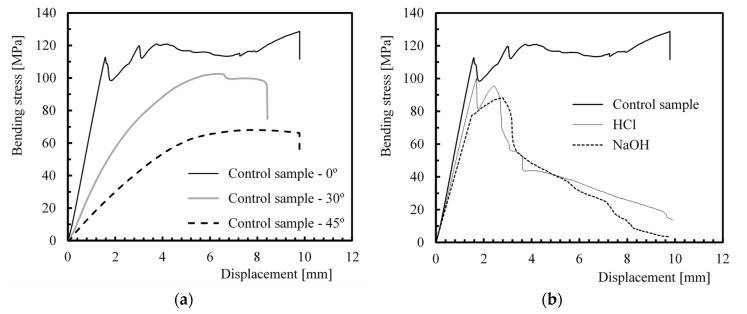
Typical flexural curves obtained for (**a**) laminates with different fibre orientations; (**b**) laminates with fibres at 0° and different hostile environments.

Three-point bending static tests were performed initially to obtain the effects of fibre orientation on the flexural properties of continuous glass fibre-reinforced polyamide 6 (Tepex^®^ dynalite 102-RG600(x)/47% Roving Glass—PA6 Consolidated Composite Laminate). In this context, Figure 1 presents typical bending stress–displacement curves, which are representative of the results obtained by other specimens.

From Figure 1a, it is possible to observe a linear increase of the load with the displacement, followed by a nonlinear behaviour, wherein the maximum load is reached. For laminates with 0° oriented fibres, the fibre breakage is the main failure mode identified, and the posterior data zigzag corresponds to the initiation of delamination’s propagation. For the other orientations, the failure mode is essentially based on matrix and/or fibre/matrix interface degradations that are evidenced by smoother curves [51]. However, the high stress concentration in the pin load contact region should not be neglected in all failure mechanisms [52]. Similar behaviour is observed when the samples were exposed to the different hostile solutions, as shown in Figure 1b, for the composites with a fibre orientation of 0°, although the data showed lower values of maximum bending stress and flexural modulus. The quantitative results presented in Table 2 are the average values of these properties for all conditions analysed.

The results show that flexural strengths of the control specimens with fibres at 30° and 45° were about 32.9% and 49.7%, respectively, lower than that of the control specimens with fibres at 0°. In terms of the bending modulus, the values decreased around 54.2% and 68.2%, respectively.

However, when the samples were immersed in hostile solutions, these decreases were more expressive. The 0° oriented fibres when exposed to acid and alkaline solutions showed values of flexural strength that were around 24.5% and 29.6% lower than that of the control group with the same fibre orientation, and the bending modulus decreased to about 11.2% and 18.7%, respectively. Physical and chemical reactions promoted by exposure to these solutions affect the matrix (chains scission) and the interfacial fibre/matrix bond strength [19,20,21,22,23], which accelerates the failure mechanisms described above and, consequently, changes its mechanical properties. While the physical reactions caused embrittlement, the chemical one produced irreversible degradation of the molecular structure [17]. According to Han and Nairn [4], polyimide laminates can microcrack spontaneously when exposed to hostile environments. However, the worst results were obtained with the alkaline solutions, demonstrating its higher severity, which agrees with the results reported in the open literature [11,13,45,53].

**Figure 2:**
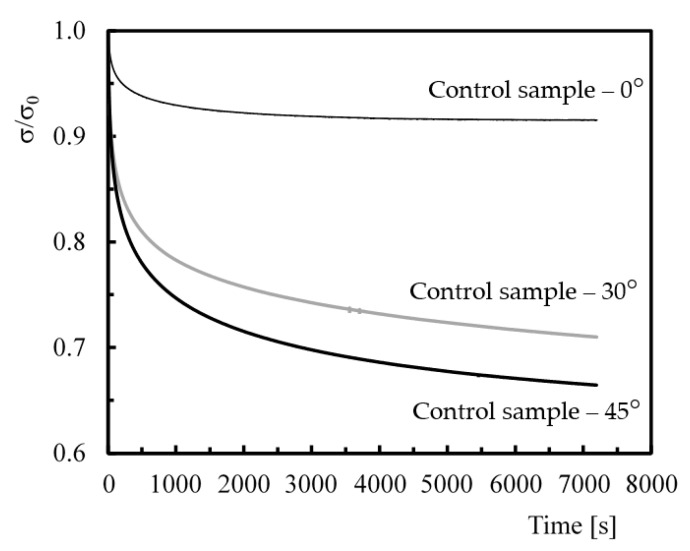
Sensitivity of stress relaxation of different fibre orientations.

For the stress relaxation, Figure 2 shows typical curves obtained for control samples and different fibre orientations. This figure plots the average bending stress versus time, where *σ* is the bending stress at any given moment of the test and *σ*_0_ is the initial bending stress. These results are representative of the stress relaxation behaviour of all conditions analysed.

It is possible to conclude that, independently of the fibre orientation, the stress decreases with time, but laminates with higher angles are more prone to stress relaxation. Notice that, when fibres angle increased, the percentage of the load that was supported by fibres decreased, and therefore, this phenomenon is mainly controlled by the matrix behaviour. For example, while the difference between initial and final stresses (after 7200 s) was about 10.3% for the laminates with fibres at 0°, this value increased to 30.3% for fibres at 30° and to 35.7% for fibres at 45%, evidenced by the higher effect of the matrix on the relaxation process. This strong effect of the fibre orientation on the stress relaxation behaviour agrees with the study by Kawai et al. [38]. In fact, fibres generally slow the relaxation process, because they hinder the molecular flow in the matrix [24]; however, this phenomenon depends considerably on the interface properties. Therefore, for laminates with fibres at 0°, relaxation occurs due to the initiation and propagation of the fibre/matrix debonding process, but the effect of the polymeric matrix should not be neglected. In this case, there are essentially two mechanisms that led to stress relaxation: physical stress relaxation due to molecular rearrangements requiring little primary bond formation or breakage; and chemical stress relaxation due to chain scission, crosslink scission or crosslink formation [42,54]. In terms of laminates with fibres at 30° and 45°, in addition to the mechanisms mentioned above, the breakage of fibre/matrix bonds and their propagation would also be responsible for the relaxation process. Consequently, higher relaxation values are observed with the respective fibre angle increase.

In Figure 2, it is also possible to identify an initial regime, in which the stress decreases considerably in relation to the remaining time [26,40,45,55]. For example, considering laminates with fibres at 0°, a decreasing around 8.7% occurred after 1000 s, while the remaining decrease was only 1.5%. These values were around 22.9% and 9.3% for the 30° fibre orientation and were 27.7% and 11% for the orientations at 45°, respectively.

**Figure 3:**
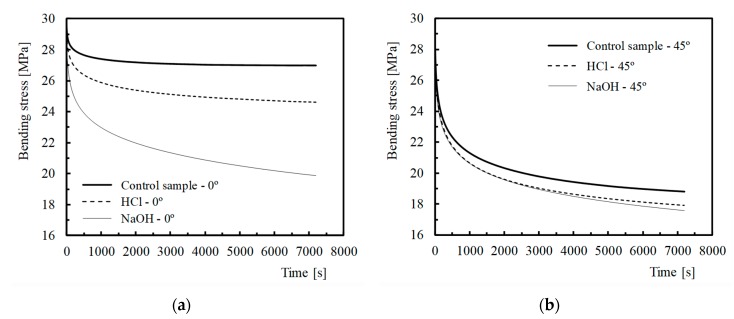
Stress relaxation curves for (**a**) different hostile solutions and laminates with fibres at 0°; (**b**) different hostile solutions and laminates with fibres at 45°.

The sensitivity to hostile environments (acid and alkaline solutions) is shown in Figure 3 for the laminates with fibres at 0° and 45°. These curves plot the average bending stress versus time and are representative of all conditions analysed, where *σ* is the time-dependent bending stress and *σ*_0_ is the initial bending stress. Regardless of the hostile solution, the stress always decreased over time, but the decreases were higher for the exposed specimens than those observed for the corresponding control samples. Table 3 presents all the stress relaxation results, where Δ*σ* is the ratio of the difference between initial and final stress.

For example, exposures to HCl solutions were responsible for an average stress decrease of around 18%, 34% and 40.3%, for the fibre orientations of 0°, 30° and 45°, respectively. These values were 74.8%, 12.2% and 12.9% greater than those verified for the corresponding control samples, i.e., for the control specimens with fibre orientations of 0°, 30° and 45°. Nevertheless, for alkaline solutions these values were higher and reached values of about 33.7%, 37% and 42%, which correspond to increases of 227.2 %, 22.1% and 17.6%, respectively.

In terms of solutions, both were responsible for higher relaxations, but the highest stress relaxations were achieved in alkaline environments, with values 3.3 times higher for laminates with fibres at 0° and around 1.2 times higher for 45°. The lower mechanical properties that were conveniently discussed above explain this behaviour. In fact, exposure to corrosive fluids significantly compromises the load carrying capacity, due to the lower mechanical properties of the matrix and the lower fibre/matrix bond strength [11,13,53]. Studies developed by Hirai et al. [56] showed, for example, that PA6 is easily plasticized when subjected to wet conditions, which significantly affects its mechanical performance. Other evidence from Table 3 and Figure 3 is the higher effect of the hostile environments on the laminates with fibres at 0° (74.8% for HCl and 227.2% for NaCl solutions) than in the other orientations. This is a consequence of the physical and/or chemical reactions between fibres and matrix [19], which affect the interfacial fibre/matrix bond strength and, consequently, higher stress relaxations. When solutions penetrate through microcracks or interface fibre/matrix, they react with the resin/fibre interface, promoting its debonding [18,19,21,22]. However, although this phenomenon also occurred in the other orientations, it was more evident in laminates with fibres of 0° due to the higher load carrying capacity.

In order to predict the stress relaxation response, the literature reports several models based on spring-dashpot systems or on more complex formulations. While the first ones failed, because the data were not fitted by a linear function, the Kohlrausch–Williams–Watts (KWW) function has been suggested in many studies to obtain more accurate predictions [26,40,45,57,58,59,60,61,62]. This relaxation function, ∅, is time dependent and is given by Equation (3):

(3) $$\emptyset \left(t\right)=\frac{\sigma \left(t\right)}{\sigma_{0}}=e^{-{\left(\frac{t}{\tau }\right)}^{\beta }}$$

where *σ*(*t*) and *σ*_0_ are the stress at time *t* and at *t* = 0, respectively, *β* is a fractional power exponent (known as non-exponential factor) and *τ* is the KWW relaxation time.

**Figure 4:**
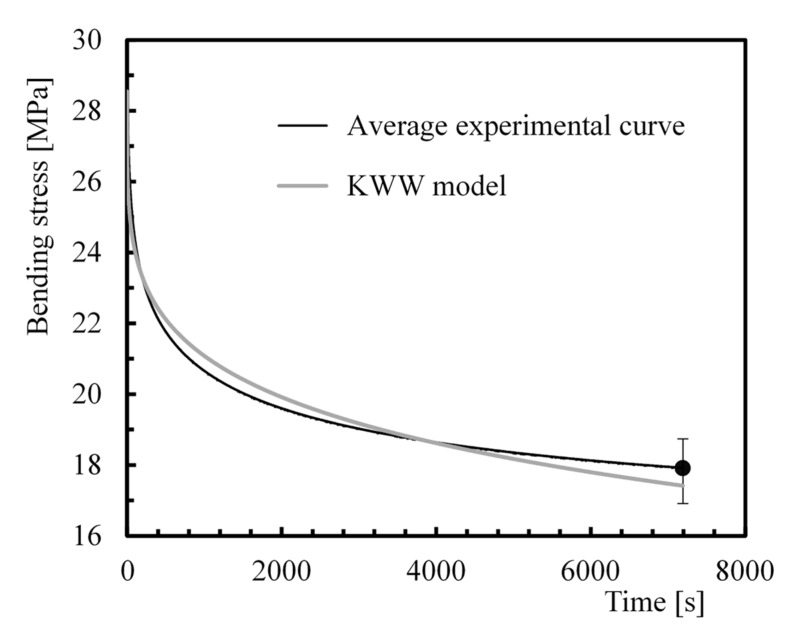
Comparison between the experimental average curve and theoretical curve obtained with the Kohlrausch–Williams–Watts (KWW) model for laminates with fibres at 45° exposed to acid solutions (HCl).

The average experimental curves and theoretical ones obtained with the KWW model are compared in Figure 4, which is representative of the results of laminates with fibres at 0°, but it is also illustrative of comparisons for all fibre orientations. The final bands represent the maximum and minimum experimental values obtained. Table 4 presents all parameters of the KWW model and respective error obtained after 7200 s, between both curves (experimental and theoretical curves). It is possible to conclude that the Kohlrausch–Williams–Watts function fits the data successfully, because the maximum error obtained in all conditions is 2.93%.

## 4. Conclusions

This work intended to study the stress relaxation behaviour of glass/polyamide 6 composites, and for this purpose, different fibre directions and exposition to different hostile environments were analysed. NaOH and HCl solutions were selected to understand the effect of alkaline and acidic environments on the long-term behaviour of such materials.

From the static bending tests, it was concluded that the flexural strength and modulus decreased by increasing the fibre orientation angle. Compared with the laminates with fibres at 0°, flexural strength decreased around 32.9% and 49.7%, respectively, for the orientations of 30° and 45°. However, exposure to hostile solutions promoted significant decreases.

Regardless of fibre orientation, stress decreased over time, but laminates with higher angles were more prone to stress relaxation. While the difference between initial and final stress was around 10.3% for laminates with fibres at 0°, this value increased to 35.7% for laminates with fibres at 45%. However, these values increased to 18% and 40.3% for HCl solutions and 33.7% and 42% for alkaline solutions, respectively. Finally, the Kohlrausch–Williams–Watts equation was used to predict the stress relaxation time, and good accuracy was obtained between the experimental and theoretical results.

## Figures and Tables

**Table 1:** Hostile environments.

Solutions	pH Level	Fibre Orientation	Immersion Time (Days)
Control sample (CS)	-	0°	-
		30°	
		45°	
HCl	0.6	0°	30
		30°	
		45°	
NaOH	13.7	0°	30
		30°	
		45°	

**Table 2:** The effects of different solutions and exposure time on bending properties.

Samples		Flexural Strength/MPa		Flexural Modulus/GPa	
		Average Value	Std Dev	Average Value	Std Dev
CS	0°	152.2	12.1	10.7	0.7
	30°	102.1	10.4	4.9	0.3
	45°	76.5	2.8	3.4	0.2
HCl	0°	114.9	13.3	9.5	0.8
	30°	95.9	3.9	3.6	0.3
	45°	66.2	5.0	3.1	0.2
NaOH	0°	107.1	12.2	8.7	0.3
	30°	92.9	7.1	3.1	0.4
	45°	62.1	2.9	2.8	0.7

**Table 3:** Effect of the hostile solutions and fibre orientations on the stress relaxation behaviour.

Samples		Initial Stress/MPa	Final Stress/MPa		Δ*σ*/%
			Average Value	Std Dev	
CS	0°	30	26.9	0.46	10.3
	30°		20.9	0.81	30.3
	45°		19.3	0.45	35.7
HCl	0°	30	24.6	1.12	18.0
	30°		19.8	1.44	34.0
	45°		17.9	1.46	40.3
NaOH	0°	30	19.9	1.31	33.7
	30°		18.9	1.13	37.0
	45°		17.4	1.10	42.0

**Table 4:** Parameters of the KWW model for stress relaxation.

Fibre Orientation	Solution	*β*	*τ*	Bending Stress after 3 h (MPa)		
				Exp. Value	KWW Value	Error (%)
0°	Control	0.173419	5,451,440,860	26.98	26.79	0.71
	HCl	0.210335	17,397,543	24.61	24.38	0.94
	NaOH	0.245194	254,842	19.87	19.71	0.81
30°	Control	0.214439	891,560	20.98	20,7	1.35
	HCl	0.221025	381,474	19.76	19.37	2.01
	NaOH	0.22324	245,827	18.92	18.58	1.83
45°	Control	0.224938	304,551	19.30	18.88	2.22
	HCl	0.247563	124,048	17.92	17.41	2.93
	NaOH	0.252633	116,068	17.58	17.24	1.97

## Nomenclature

*b*	Width of the specimen (mm)
*h*	Thickness of the specimen (mm)
*I*	Moment of inertia of the cross-section (mm^4^)
*L*	Spam length (mm)
*P*	Load (N)
*t*	Time (seconds)
*β*	Fractional power exponent (known as non-exponential factor)
Δ*P*	Load range in the middle span for an interval in the linear region (N)
Δ*u*	Flexural displacement range in the middle span for an interval in the linear region (mm)
Δ*σ*	Difference between initial and final stress divided by the initial stress
*σ*_0_	Stress at *t* = 0; initial bending stress
*σ*(*t*)	Stress at time *t*
*σ*	Bending stress at any given moment of the test
*τ*	KWW relaxation time
∅	Relaxation function

## Abbreviations

ASTM	American Society for Testing and Materials
GFRP	Glass fibre-reinforced polymer
GRP	Glass-reinforced pipes
H^+^	Hydrogen ions
H_2_O	Water molecule
HCl	Hydrochloric acid
HNO_3_	Nitric acid
H_3_PO_4_	Phosphoric acid
H_2_SO_4_	Sulfuric acid
NaCl	Sodium chloride
NaOH	Sodium hydroxide
PA6	Polyamide 6
KWW	Kohlrausch–Williams–Watts function
SR	Stress relaxation
Std Dev	Standard deviation
3PB	Three-point bending static tests
